# Renal Dnase1 Enzyme Activity and Protein Expression Is Selectively Shut Down in Murine and Human Membranoproliferative Lupus Nephritis

**DOI:** 10.1371/journal.pone.0012096

**Published:** 2010-08-10

**Authors:** Svetlana N. Zykova, Anders A. Tveita, Ole Petter Rekvig

**Affiliations:** 1 Department of Biochemistry, Institute of Medical Biology, Medical Faculty, University of Tromsø, Tromsø, Norway; 2 Department of Rheumatology, University Hospital of Northern Norway, Tromsø, Norway; Institut Jacques Monod, France

## Abstract

**Background:**

Deposition of chromatin-IgG complexes within glomerular membranes is a key event in the pathogenesis of lupus nephritis. We recently reported an acquired loss of renal *Dnase1* expression linked to transformation from mild to severe membranoproliferative lupus nephritis in (NZBxNZW)F1 mice. As this may represent a basic mechanism in the progression of lupus nephritis, several aspects of Dnase1 expression in lupus nephritis were analyzed.

**Methodology/Principal Findings:**

Total nuclease activity and Dnase1 expression and activity was evaluated using *in situ* and *in vitro* analyses of kidneys and sera from (NZBxNZW)F1 mice of different ages, and from age-matched healthy controls. Immunofluorescence staining for Dnase1 was performed on kidney biopsies from (NZBxNZW)F1 mice as well as from human SLE patients and controls. Reduced serum Dnase1 activity was observed in both mesangial and end-stage lupus nephritis. A selective reduction in renal Dnase1 activity was seen in mice with massive deposition of chromatin-containing immune complexes in glomerular capillary walls. Mice with mild mesangial nephritis showed normal renal Dnase1 activity. Similar differences were seen when comparing human kidneys with severe and mild lupus nephritis. Dnase1 was diffusely expressed within the kidney in normal and mildly affected kidneys, whereas upon progression towards end-stage renal disease, Dnase1 was down-regulated in all renal compartments. This demonstrates that the changes associated with development of severe nephritis in the murine model are also relevant to human lupus nephritis.

**Conclusions/Significance:**

Reduction in renal Dnase1 expression and activity is limited to mice and SLE patients with signs of membranoproliferative nephritis, and may be a critical event in the development of severe forms of lupus nephritis. Reduced Dnase1 activity reflects loss in the expression of the protein and not inhibition of enzyme activity.

## Introduction

Systemic lupus erythematosus (SLE) is a systemic autoimmune disease characterized by the development of autoreactivity against nuclear antigens, including double-stranded DNA (dsDNA) and histones [1], [2], [3]. The predominance of chromatin-associated antigen targets points at aberrancies in the processing and elimination of chromatin as a potential culprit of such a process [4], [5], [6], [7], [8]. It has been postulated that effective degradation of DNA from dying cells is essential to prevent priming of the immune system against chromatin self-antigens, and impaired chromatin degradation has been proposed as a mechanism for the development of antinuclear autoimmunity [9], [10].

DNA fragmentation by the activation of various nucleases is considered a key event in apoptotic cell death (reviewed in [11], [12]). For elimination of DNA from necrotic cells, secreted nucleases, including Dnase1 are assumed to play a central role in this process (reviewed in [11], [13]). Under circumstances of increased cellular stress, such as active infections, malignancies and tissue trauma, increased amounts of DNA can be observed within the circulation, suggesting that the capacity for DNA elimination is exceeded [14], [15], [16]. Increased levels of circulating DNA and nucleosomes have been reported in SLE [17], [18], [19], especially in active stages of the disease [20] and in lupus-prone mice [21].

Dnase1 is considered the major serum nuclease, and has been a topic of interest in the context of SLE for several decades. Dnase1 is the founding member of the Dnase1-like (Dnase1l) family of divalent cation-dependent endonucleases, which also include Dnase1l1-3. Reduced serum Dnase1 activity is a common finding in SLE patients [22], [23], [24] and lupus-prone mice [25]. The basis for increased concentration of DNA in the circulation remains controversial [13], but possible explanations include ineffective elimination of chromatin due to impaired nuclease activity, either by decreased nuclease availability [23] or inhibition by factors such as actin [22], [26], [27]. Attempts at Dnase1 enzyme replacement therapy in mice and SLE patients have been largely disappointing [28], [29], as has experimental over-expression of Dnase1 in T-cells in lupus-prone mice [30]. In contrast, experimental deletion of *Dnase1* in mice resulted in development of lupus-like disease, including anti-chromatin autoantibody production and immune-complex mediated glomerulonephritis [31]. Later studies revealed that these effects were largely eliminated upon backcrossing into one of the parental strains, suggesting that other predisposing genetic aberrancies are required for the development of autoreactivity in this model. The data, however, suggest that eliminating Dnase1 contributes to the acceleration of renal disease in lupus-prone mice [13].

Taken together, these data suggest that Dnase1 deficiency alone is not sufficient to induce autoimmunity against chromatin, but may play a key role in progression of lupus nephritis. Whereas the activity of Dnase1 in serum has been extensively studied [22], [23], [25], [33], little is known about its expression and activity within different organs. In a recent study, we demonstrated that the appearance of anti-dsDNA antibodies in (NZBxNZW)F1 (B/W) mice coincided with early signs of development of mesangial nephritis, while reduced renal Dnase1 mRNA expression correlated with progression of lupus nephritis into end-stage organ disease [32]. This recent data is indicative of loss of DNase1 serving as a possible factor in the progression of lupus nephritis.

The present study was designed to further characterize these changes in terms of *i.* localization of renal Dnases in different compartments of the kidneys, *ii.* their relative contribution to total renal nuclease activity and *iii.* the relation between renal and serum Dnase1 activity with initiation and progression of lupus nephritis. Furthermore, to evaluate whether changes in Dnase1 expression could also be involved in the progression of the human variant of lupus nephritis, renal Dnase1 expression was also assayed in biopsies from SLE patients and correlated with morphological and immune electron microscopy findings.

## Results

### Characteristics of the experimental animals

High anti-dsDNA antibody titer was present in all the animals with nephritis irrespective of age (26–38 w.o.) and was elevated for an average of 2 months prior to the development of overt proteinuria [34]. Young B/W mice and BALB/c control mice of all age groups had no clinical or laboratory signs of kidney disease. Pre-proteinuric animals on average had only marginally elevated anti-dsDNA antibody titers. The predominant pattern of glomerular immune complex (IC) deposits was characterized by immune electron microscopy (representative images are presented in Figure S1). Based on these results, the proteinuric mice were divided into two groups, one with mesangial IC deposits (Figure S1A–B), and one with IC deposits in the glomerular capillary walls (Figure S1C–D). The pre-nephritic mice had no glomerular deposits (Figure S1E–F). Data presented below demonstrate the relevance of these groups in identifying pathogenic factors in lupus nephritis.

Gene expression data for major apoptotic nucleases in the kidneys from mice of different ages is presented in Table 1. The results are expressed as fold change relative to 4 weeks old BALB/c control mice and show that of all the renal nucleases studied, only the mRNA level of *Dnase1* was significantly reduced in the group of mice with membranoproliferative nephritis. Lack of down-regulation of other genes, including house-keeping genes or genes encoding other renal nucleases, makes it unlikely that reduced Dnase1 expression was due to renal insufficiency or loss of viable renal cells (see below).

**Table 1:** Gene expression of apoptotic nucleases in kidneys from (NZBxNZW)F1 and BALB/c mice from different age groups.

Taqman assay	Mouse	Age			
		4 w.o.	8 w.o.	20 w.o.	>26 w.o.
*dnase1*Mm01342389_g1	B/W	1.15±0.09^a^	1.82±0.18	1.69±0.42	**0.45±0.59^f^**
	BALB/c	1.07±0.42	1.59±0.27	1.30±0.49	1.49±0.30
*dnase2*Mm00438463_m1	B/W	0.82±0.10	0.81±0.18	1.38±0.11	1.43±0.19
	BALB/c	1.01±0.18	1.19±0.23	1.00±0.05	1.16±0.22
*endoG* bMm00468248_m1	B/W	0.88±0.15	0.78±0.19	1.00±0.11	0.76±0.23
	BALB/c	1.00±0.06	1.18±0.20	1.04±0.17	0.98±0.03
*Dffa* cMm00438410_m1	B/W	1.09±0.14	0.81±0.12	0.94±0.08	1.24±0.14
	BALB/c	1.00±0.09	1.14±0.24	0.98±0.16	0.86±0.08
*Dffb* dMm00432822_m1	B/W	0.84±0.10	0.74±0.13	0.89±0.09	0.87±0.13
	BALB/c	1.00±0.09	0.78±0.16	0.81±0.03	0.68±0.20
*Cideb* eMm00438213_m1^g^	B/W	1.22±0.23	1.04±0.15	1.48±0.33	1.09±0.52
	BALB/c	1.01±0.11	1.28±0.22	1.00±0.17	1.16±0.15

a The data are presented as fold change relative to an average level of the 4 w.o. BALB/c mice (mean ± SEM, n = 5), after normalization for the expression level of a house-keeping gene in the same sample (Mouse ACTB (actin, beta) Endogenous Control (4352933E) and TATA-box binding protein (Mm00446973_m1).

b endonuclease G.

c DNA fragmentation factor, alpha subunit.

d DNA fragmentation factor, beta subunit.

e cell death-inducing DNA fragmentation factor, alpha subunit-like effector B.

**^f^**significantly reduced *Dnase1* gene expression only in kidneys from proteinuric B/W mice.

g Real time PCR was performed on ABI Prism 7900HT Sequence Detection System with the TaqMan Gene Expression Assay indicated for each gene.

### Total renal and serum nuclease activity in progressive murine lupus nephritis

**Figure 1:**
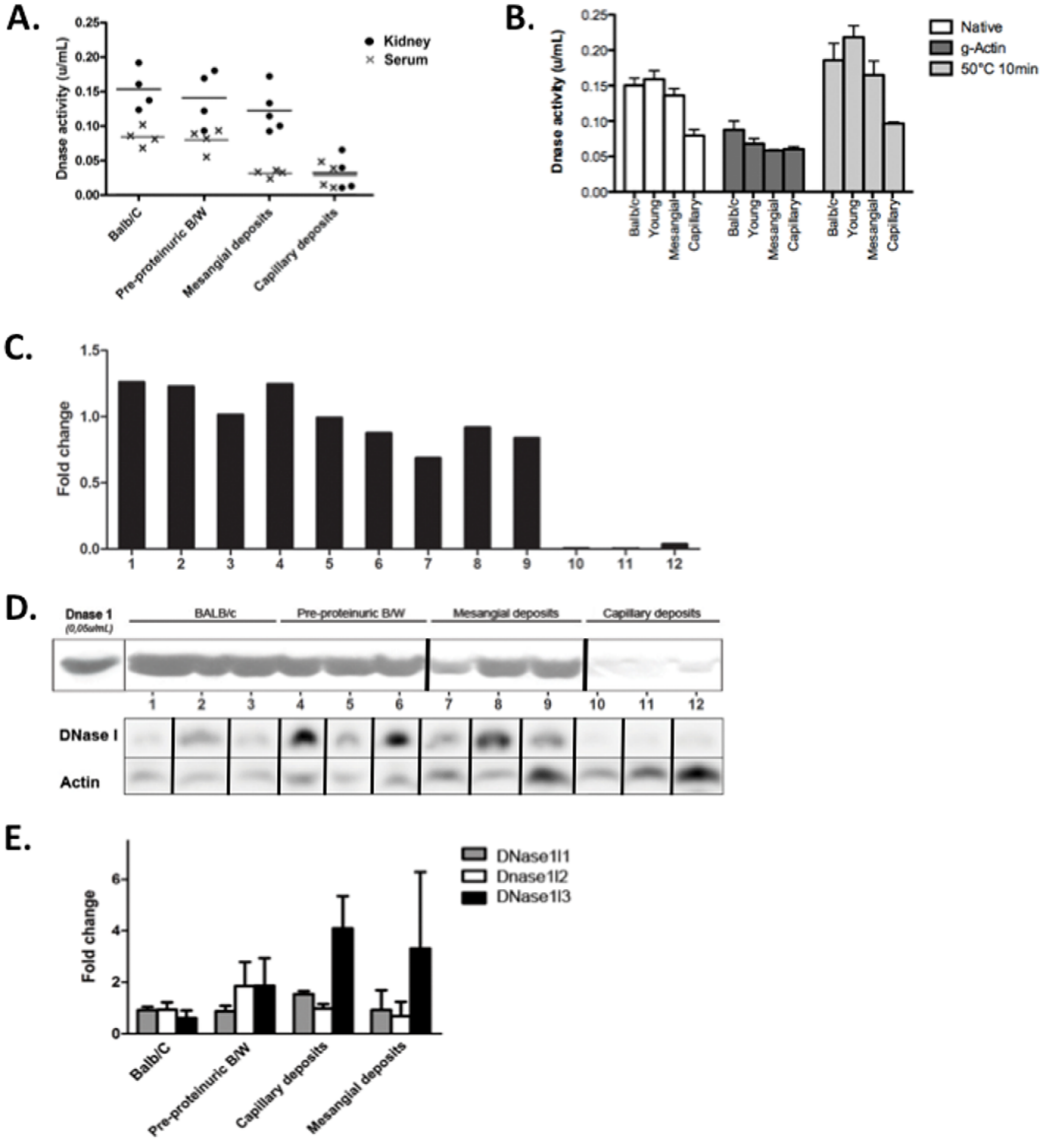
Total nuclease activity, Dnase1 and Dnase1l1-3 expression in kidneys from BALB/c and (NZBxNZW)F1 mice. Pre-proteinuric and proteinuric B/W kidneys were used, the latter divided into 2 groups; one characterized by immune complex deposits in glomerular mesangial matrix only (labeled “Mesangial deposits”), and the other with immune complex deposits in both the mesangial matrix and the capillary membranes (labeled “Capillary deposits”). (*A*) Kidney lysates (black) or sera (grey), in which total protein was measured, and protein content of the samples normalized using BCA assay (Pierce), were applied onto 1% agarose gels containing calf thymus DNA and ethidium bromide in a CaCl_2_- and MgCl_2_- containing buffer, pH 7.6, and incubated in a humidified chamber at 37°C for 24 hours. The gel was photographed under UV illumination. The radius of the non-fluorescent area surrounding each well reflected DNase activity in the sample. DNase activity is expressed as Dnase1 equivalent units, by comparison with human recombinant Dnase1. (B) Total nuclease activity in renal homogenates is shown for native samples, after addition of 5µg/mL monomeric actin and after preheating the samples to 50°C for 10 minutes prior to incubation on the gel, reflecting the results of addition and reversal of actin inhibition, respectively. The group labeled “young” represent 10 and 20 week old B/W, whereas the “Mesangial” and “Capillary” groups reflect proteinuric B/W with immune complex deposits in the mesangium and GBM, respectively. DNase activity is expressed as Dnase1 equivalent units, by comparison with human recombinant Dnase1. In panels C and D the order of samples are kidneys from normal age matched BALB/c (lanes 1–3), pre-nephritic B/W kidneys (lanes 4–6), nephritic B/W kidneys with mesangial matrix deposits only (lanes 7–9), and nephritic B/W kidneys with capillary membrane deposits (lanes 10–12). All kidney samples were analyzed for fold change of renal *Dnase1* mRNA levels relative to 4 weeks old BALB/c mice (C), zymography of Dnase1 activity in 10% SDS-polyacrylamide gel of kidney lysates (D, upper part), and western blot of Dnase1 in the same samples (D, lower part). Western blotting to detect actin in the same samples demonstrated that the low Dnase1 band intensity in lanes 10–12 was not due to unequal loading of the samples on the gel. Renal mRNA expression of Dnase1l1 (TaqMan assay Mm00510102_m1), Dnase1l2 (Mm00481868_g1) and Dnase1l3 (Mm00432865_m1) was not decreased in kidneys from proteinuric mice (E).

In order to evaluate the Ca^2+^- and Mg^2+^-dependent DNase activity in kidneys and sera, kidney homogenates and sera from individual B/W and BALB/c mice of different ages were analyzed by single radial enzyme diffusion (SRED) assay. This allows evaluation of net DNA degrading activity, taking into account the effects of any Dnase1 inhibitory factors present within the samples. There was a significant reduction in total nuclease activity in the proteinuric B/W kidneys compared to those of both younger B/W and age-matched BALB/c mice (Figure 1A). Importantly, the loss of nuclease activity was only observed in mice with glomerular capillary membrane deposits of chromatin fragment-IgG complexes (p<0.001), while nuclease activity was normal in kidneys with deposits confined to the mesangium (Figure 1A). Corresponding assays for total serum nuclease activity revealed a less pronounced but consistent decrease in the proteinuric mice regardless of the pattern of glomerular chromatin-IgG deposits (Figure 1A, p<0.001). No evidence of decreased serum nuclease activity was seen in pre-nephritic mice or in B/W mice prior to anti-dsDNA antibody production. Dnase1 differs from other members of the Dnase1-like family of nucleases by its sensitivity to inhibition by monomeric actin. Pre-incubating the gel in buffer containing 5mM actin caused a marked reduced serum and renal SRED activity in mice of all age groups, with similar residual Dnase activity in proteinuric and non-proteinuric mice (Figure 1B). These data suggest that the observed reduction in serum and renal Dnase activity is caused by the loss of one or more actin-inhibitable factors. Preheating the samples to 56°C for 10 minutes, previously reported to reverse Dnase1 inhibition by actin [22], [35], caused a proportionally similar increase in SRED activity in both serum and renal homogenates in mice of all ages (Figure 1B), suggesting that inhibition of Dnase1 by actin does not explain the observed differences in nuclease activity in these mice. Inhibition of the actin-resistant Dnase1 homologue Dnase1l3 using the selective inhibitor DR396 showed no differences in Dnase activity by the SRED assay in any mice (data not shown). Gel zymography of sera showed enzyme activity confined to a single band corresponding in size to Dnase1, which was completely inhibited by incubating the gel in the presence of actin (data not shown). While serum nuclease activity was reduced in mesangial and membranoproliferative nephritis, renal Dnase1 activity was reduced only in membranoproliferative nephritis. These results suggest that changes in renal nuclease activity are due to changes intrinsic to the kidney, and not a passive reflection of serum DNase activity. The fact that there is a parallel loss of renal Dnase1 mRNA and the Dnase1 protein (Figure 1C), argue that the loss of Dnase1 in the kidney is a consequence of shut-down of gene expression.

### Dnase1 expression and zymography

To evaluate how reduced renal *Dnase1* gene expression affects enzymatic activity in the proteinuric mice, Dnase1 activity in kidney homogenates was assayed after electrophoretic separation of proteins in DNA-containing gels. Renal Dnase1 mRNA expression levels in the individual samples were compared with zymographic renal Dnase1 activity in the same mice (Figure 1C and D for gene expression and zymography, respectively). Zymography revealed DNA-degrading activity predominantly in a band corresponding to the molecular weight of recombinant human Dnase1 (Figure 1D, lanes 1–3 for BALB/c, lanes 4–6 for pre-nephritic B/W mice, and lanes 7–12 for nephritic B/W mice). Activity in this band was completely eliminated in all samples when incubation of the gel was performed in the presence of actin (data not shown), further identifying the responsible enzyme as Dnase1. The samples obtained from nephritic B/W mice clearly separated into two distinct groups: those with mild nephritis and immune complex deposits confined to the mesangial matrix demonstrated normal levels of Dnase1 mRNA and Dnase1 enzyme activity (Figure 1C and D, lanes 7–9), whereas mice with severe nephritis and IC deposits within the capillary membrane showed dramatically reduced Dnase1 mRNA and enzyme activity (Figure 1C and D, lanes 10–12). Thus, there was a consistent correspondence between levels of *Dnase1* mRNA expression and Dnase1 activities within the same kidneys. Several weak bands of high molecular weight (100–150kDa) with DNA-degrading activity were also observed, with similar intensity in all samples from both pre-nephritic and nephritic kidneys (data not shown).

Renal mRNA expression of the Dnase1 homologues Dnase1-like 1, -2, -3 was also assayed in the various groups of mice. The results demonstrated no evidence of significant nephritic stage reduction or compensatory up-regulation of any of these nucleases (Figure 1E, p = 0.1768 for Dnase1l1, p = 0.1497 for Dnase1l2, p = 0.0780 for Dnase1l3). A tendency for gradual elevation of Dnase1l3 expression with nephritis progression was observed, however, no formal statistical significance was reached when either parametric (ANOVA) or non-parametric (Kruskal-Wallis) tests were applied.

### Detection of renal Dnase1 protein by western blotting

In order to clarify whether the low Dnase1 activity in the kidney homogenates from B/W mice with membranoproliferative nephritis was due to inhibition of enzyme activity or to an absolute reduction in renal Dnase1 protein amount, western blotting was performed on the same samples that were used for SDS-PAGE gel zymography (Figure 1D). A single band was seen, corresponding to the MW of recombinant human Dnase1 (rhDnase1, approximately 32 kDa, Figure 1D). Very low band intensities were found in the kidney samples that demonstrated considerably reduced *Dnase1* mRNA levels and Dnase1 enzyme activity (Figure 1D, lanes 10–12), whereas mice with normal mRNA expression and mesangial matrix deposits only, had Dnase1 protein expression comparable to the normal control and pre-proteinuric mice (Figure 1D, lanes 7–9). Immunoblotting to detect actin in the same samples demonstrated that the low Dnase1 band intensity in lanes 10–12 could not be explained by unequal loading of the samples on the gel. (Figure 1D). Immunoblotting against Dnase1l1 showed similar levels of expression in kidneys from B/W mice of all ages (data not shown).

### *In situ* DNA-degradation assay

**Figure 2:**
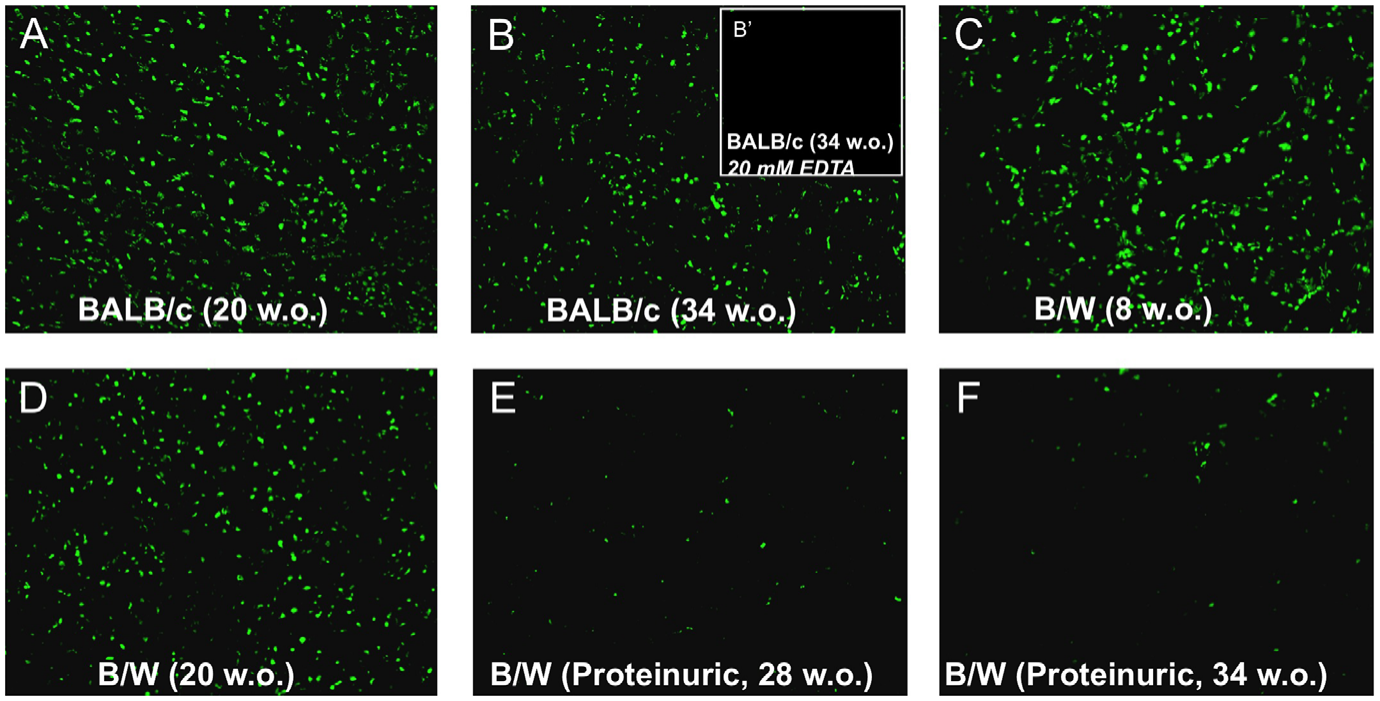
Low rate of nucleolysis in proteinuric (NZBxNZW)F1 kidneys. Non-fixed cryosections of kidneys were incubated in DNase reaction buffer (2mM CaCl_2_, 2mM MgCl_2_ in 40mM Tris, pH7.6) to allow enzymatic cleavage of endogenous DNA by nucleases present in the tissue. TUNEL-based detection of DNA fragmentation was performed after 2h incubation at 37°C. Results are given as TUNEL-stained endogenous DNA in kidney sections from 20 w.o. (A) and 32 w.o. (B) BALB/c, 8 w.o. (C), 20 w.o. (D) and proteinuric 28 w.o. (E) and proteinuric 34 w.o. (F) B/W mice. The 2 latter mice suffered from membranoproliferative nephritis with very low renal *Dnase1* mRNA levels and Dnase1 enzyme activity. Control analyses were performed by adding 20 mM EDTA to the reaction buffer. This completely abolished TUNEL staining in 34 w.o. BALB/c kidneys (see inserted panel B' in panel B) as well as in kidneys from young and proteinuric B/W mice (data not shown). Similarly, immediate fixation of the tissue sections with paraformaldehyde also resulted in absence of nicked DNA (data not shown).

To visualize areas of renal nuclease activity in situ, non-fixed cryosections of kidneys were incubated in DNase reaction buffer to allow enzymatic cleavage of endogenous nuclear DNA by nucleases present in the tissue. The DNA nicks generated by endogenous nucleases were identified by terminal deoxynucleotidyl transferase-mediated incorporation of dUTPs labeled with a fluorescent marker. The signals generated were present in nuclei of renal cells in BALB/c (Figure 2A and B) and in pre-nephritic B/W (Figure 2C and D) mice, while significantly reduced intensity was observed in proteinuric B/W mice with capillary membrane chromatin-IgG deposits (Figure 2E and F). The reaction was completely blocked by EDTA (Figure 2B, insert labeled B'), confirming that the DNA degradation was divalent cation-dependent, and no signal was seen upon immediate fixation with paraformaldehyde (data not shown). These results corresponded well with data presented in Figure 1.

### Detection of the renal Dnase1 protein *in situ* by indirect immunofluorescence in murine and human forms of lupus nephritis

**Figure 3:**
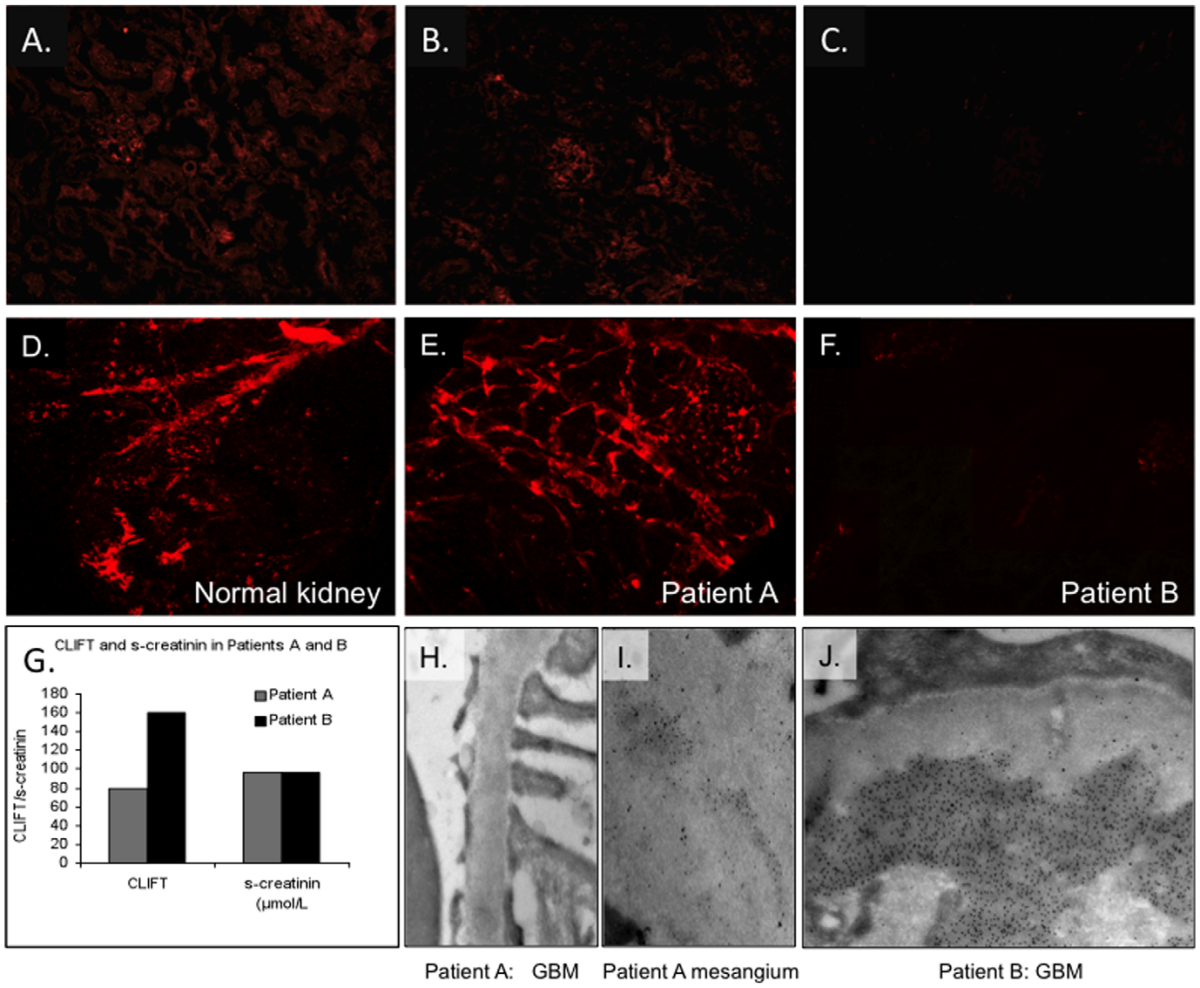
Expression of immunoreactive Dnase1 in kidneys from (NZBxNZW)F1 mice and from patients with lupus nephritis. Cryosections of B/W and human kidneys were immunostained with rabbit anti-Dnase1 antibody followed by an Alexa488-conjugated F(ab')_2_ anti-rabbit IgG antibody. The images were taken using identical exposure settings. The images were obtained at 400× magnification on kidney sections from a 32 w.o. BALB/c mouse (A), a 20 w.o. B/W mouse with mesangial nephritis (B) and a 32 w.o. nephritic B/W mouse with membranoproliferative nephritis and low renal Dnase1 activity (C). In the kidneys of the latter mouse, only traces of the Dnase1 enzyme could be detected. Similarly, there was robust Dnase1 staining in a kidney biopsy from a histologically normal kidney (D), a patient with mesangial nephritis (Patient A, panels E, H and I ), whereas only traces of Dnase1 staining was detectable in kidneys from a patient with membranoproliferative lupus nephritis (Patient B, panels F and J). Crithidia luciliae immunofluorescence titers (CLIFT) and serum creatinine levels of the two patients are shown (panel G). Immune electron microscopy using protein-A-gold conjugated rabbit anti-human IgG antibodies revealed predominantly mesangial electron-dense structures (EDS) in patient A with mild nephritis (panels H-I), whereas glomerular as well as mesangial EDS were present patient B with severe nephritis (panel J).

Tissue localization of Dnase1 and its possible contribution to the *in situ* DNA-fragmentation ability as presented in Figure 2, was analyzed by indirect immunofluorescence. For a better overview of morphology of the sections, matched phase contrasts and immunofluorescence micrographs have been included (Figure S2). Staining of normal BALB/c kidneys with an anti-Dnase1 antibody (Figure 3A) revealed diffuse intracellular staining throughout the kidney, with weaker staining within glomeruli. Similar staining was observed in B/W mice with mesangial chromatin-IgG deposits (Figure 3B). Proteinuric mice with deposits in capillary membranes had significantly reduced staining of immunoreactive Dnase1 in both tubuli and glomeruli (Figure 3C, see also Figure S2 for details) compared to healthy BALB/c controls and younger pre-diseased B/W mice. Immunofluorescence staining of Dnase1l1 showed low levels of staining with comparable intensity in kidneys from mice of all ages (data not shown).

To analyze if the down-regulation of *Dnase1* gene expression and corresponding Dnase1 enzyme activity observed in nephritic BW mice was also relevant in human lupus nephritis, Dnase1 immunostaining was performed on histologically normal human kidney biopsies (Figure 3D) and to biopsies from patients with mild (Patient A, Figure 3E) and membranoproliferative (Patient B, Figure 3F) lupus nephritis. The data revealed a dramatic decrease in Dnase1 immunostaining only in renal sections from the patient with severe nephritis. For a better overview of morphology of the sections, matched phase contrast and immunofluorescence micrographs have been included for biopsies from patients A and B, respectively (Figure S3A–B). This figure demonstrates that the Dnase1 protein is lost both in tubular and glomerular cells in the latter kidney biopsy (Figure S3B). *Crithidia luciliae* immunofluorescence titer (CLIFT) and serum creatinine levels for Patients A and B are shown in Figure 3G. Immune electron microscopy, where in situ bound IgG was detected by rabbit anti-human IgG antibody followed by binding of gold-labeled protein A [36], showed chromatin-IgG deposits predominantly in the mesangial matrix of Patient A (Figures 3H–I), while in the biopsy from Patient B, the deposits were widely distributed in both glomerular basement membranes (GBM) and the mesangial matrix (Figure 3J). These data parallel the association between reduced renal Dnase1 expression and the occurrence of membranoproliferative lupus nephritis seen in B/W mice. Results from Dnase1 staining of additional biopsies from patients with lupus nephritis revealed a similar relationship between Dnase1 staining intensity and the glomerular loci for chromatin-IgG complex deposits (Figure S4). Moreover, Dnase1 staining in a biopsy of a Wegener's granulomatosis patient with severe pauci-immune glomerulonephritis was comparable to that of normal kidneys (Figure S4D), suggesting that the observed reduction in Dnase1 staining is specific for lupus nephritis, and not a general element of particular patterns of nephritis.

## Discussion

Dnase1 has been implicated in SLE pathogenesis through a number of studies in both mice and humans [23], [25], [37]. The kidney is one of the major loci for Dnase1 production and activity. Other organs with Dnase1 expression include salivary glands, liver and gastrointestinal tract cells [38]. In humans but not rodents, the pancreas appears to be an important source of Dnase1 [39]. The relative contribution of each of these organs to the circulating pool of Dnase1 in blood and urine remains uncertain. Dnase1 is the principal renal nuclease, reported to account for more than 80% of the total endonuclease activity within the kidney [40]. Interestingly, decreased levels of urine nuclease activity has been found in lupus-prone B/W mice, preceding the development of renal disease manifestation [25]. Since it has been shown that urinary nuclease activity correlates with its activity in kidneys [41], renal shut-down of the enzyme could be relevant to explain development of lupus nephritis, including deposition of large chromatin fragments (reviewed in [3], [42]).

Using both morphological and zymographic methods, the current work extends previous data demonstrating an acquired, selective down-regulation of renal *Dnase1* gene expression linked to progression towards end-stage lupus nephritis. The existence of several Dnase1-like enzymes with similar biochemical properties makes it important to assay Dnase activity by several methods to verify the identity of the responsible nucleases. Inhibition by actin is only seen for Dnase1 [43] and possibly Dnase1l1 [44]. Quantitative mRNA and immunofluorescence assays demonstrated no evidence of decreased levels of Dnase1l1 in the kidneys of proteinuric mice. We demonstrate here *i.* that the level of renal Dnase1 mRNA expression is reflected by enzyme activity and Dnase1 protein expression, *ii.* the relative contribution of renal Dnase1 to total renal nuclease activity, *iii.* the renal location of Dnase1 protein expression and loss of Dnase1 protein expression during progression of lupus nephritis, and finally *iv.* the relevance of decreased renal Dnase1 for progression of human lupus nephritis. The discrepancy between serum and renal nuclease activity within the proteinuric group, as well as the consistency of assays of mRNA, protein and enzymatic activity levels, strongly favors reduced renal Dnase1 synthesis as the major contributor to reduced renal Dnase1 activity.

A characteristic feature of lupus nephritis is the appearance of glomerular IC deposits observed as electron dense structures (EDS) by transmission electron microscopy [45], [46], [47], [48]. In a recent report, we correlated patterns of immune complex deposits with renal mRNA expression of *Dnase1* [32]. Whereas mesangial EDS were detectable in all mice that produced anti-dsDNA antibodies, a clear correlation was seen between reduced renal Dnase1 expression and the formation of capillary sub-endothelial and sub-epithelial deposits, and the development of membranoproliferative nephritis. Our current data confirms that these associations are indeed reflected in a kidney-specific decrease in renal Dnase1 activity. This change may represent an important factor in the progression of mesangial lupus nephritis into end-stage renal disease.

The results of the present immunofluorescence studies on renal biopsies from SLE patients suggest that changes in Dnase1 expression similar to those seen in the B/W mice are relevant to explain disease progression also in humans. Previous data have demonstrated a constitutive defect causing reduced oligonucleosomal DNA fragmentation in kidneys of B/W mice of all ages upon induction of apoptosis in kidneys of B/W mice using the topoisomerase I inhibitor camptothecin [34]. These results therefore led us to investigate whether such a defect could be explained by constitutively reduced expression of one or more of the nucleases responsible for apoptotic DNA fragmentation. In the present report we did not find evidence of decreased total nuclease activity in young B/W mice. This would suggest that the basis of such a camptothecin-related defect lies upstream in the apoptotic signaling cascade. Although not related to the reported reduction in apoptotic DNA fragmentation, there is a striking association between the fall in renal Dnase1 enzyme activity and the accumulation of chromatin fragment-containing IC within the GBM. Because Dnase1 is the major endonuclease within the kidney ([40], present results), an uncompensated reduction in renal Dnase1 activity could contribute to a reduced clearance and secondary exposure of chromatin debris within the kidney. Exposed secondary necrotic chromatin triggers pro-inflammatory signaling in phagocytes, and constitutes an important danger signal [49]. Failure of phagocytic clearance of apoptotic and/or necrotic cells has been postulated as a possible driving force for sustained anti-chromatin autoimmunity [42], [46], [50], [51]. If apoptotic chromatin is exposed, e.g. as a result of diminished renal Dnase1 activity, this could trigger renal inflammation and leukocyte infiltration, and at the same time provide a target for circulating anti-chromatin antibodies. In the face of pre-existing, florid autoreactivity, such changes could conceivably launch a rapidly progressive organ-centered immune attack, causing proteinuria and renal failure.

The fact that down-regulation of Dnase1 in the kidneys appears after initiation of anti-dsDNA antibody production indicates that loss of renal nuclease activity is not responsible for the appearance of anti-chromatin autoimmunity. Moreover, Wilber et al. have identified a mutation in the Dnase1l3 gene in the B/W mice that reduced the ability of this enzyme to fragment DNA [52]. This nuclease has been proposed to play a role during apoptotic chromatin degradation [53], [54]. Whether this defect has any impact on development of anti-chromatin antibodies and lupus nephritis has not been determined. Considering such data, and the data presented in this study, several defects in the apoptotic processing and elimination of DNA may together contribute to initiation and progression of e.g. lupus nephritis in these animals. However, inhibition of Dnase1l3 using a specific inhibitor did not significantly alter serum or renal Dnase activity in B/W or BALB/c mice of any ages, suggesting a modest contribution of this endonuclease to total nuclease activity in the circulation and within the kidneys.

Existing data are conflicting on the issue of whether a reduced total serum nuclease activity is present in pre-nephritic B/W mice [25], [52]. In the present study, no reduction in activity was found in pre-proteinuric mice. Still, the consistent decrease seen in serum nuclease activity in all proteinuric mice irrespective of the levels of renal total nuclease expression indicate that similar changes could occur in other organs, including the liver. Such changes might therefore be of relevance to the loss of immunological tolerance against DNA and nucleosomes, and is currently being analyzed in our laboratory. Furthermore, the lack of a consistent correlation between serum and renal Dnase1 activity suggests that these reflect separate processes; the latter possibly confined to the kidney and related to the development of particularly unfavorable patterns of progression of the renal disease.

Considering the widespread hypothesis that SLE is related to deficient clearance of apoptotic debris, and in particular to the removal of chromatin-associated antigens, impaired production of a key secreted renal nuclease in a spontaneous model of the disease is a striking observation. An acquired down-regulation of Dnase1 expression during the development of lupus nephritis could be relevant to understand how systemic autoimmunity translates into end-organ disease, and could prove useful as a clinical marker for renal disease in SLE, as discussed by Mortensen et al. [42]. Identifying the processes underlying the observed down-regulation of renal Dnase1 is a complex feat, and is the focus of ongoing investigations.

## Materials and Methods

### Ethics Statement

The National Animal Research Authority (NARA) approved the study. Treatment and care of animals were conducted in accordance with guidelines of the Norwegian Ethical and Welfare Board for Animal Research. The study was approved by the Regional Ethical Committees in Lund, Sweden, and in Northern Norway.

### Collection of samples from B/W and BALB/c mice

Female B/W and BALB/c mice were purchased from Harlan (Blackthorn, UK). Serum samples were collected every second week. Proteinuria was monitored weekly with sticks from Bayer Diagnostics (Bridgend, UK). Staining of ≥3+ (≥3 g/L) was regarded as proteinuria indicative of overt nephritis. Kidneys were extirpated from B/W mice of different ages and from gender- and age-matched BALB/c control mice (Table 1), cut and snap-frozen in liquid N_2_ with or without OCT (Optimal Cutting Temperature, Tissue-Tek, Terrance, CA), fixed in buffered depolymerised paraformaldehyde for electron microscopy studies, or stored in RNAlater (Ambion Inc, Austin, TX) for studies of gene expression. Treatment and care of animals were conducted in accordance with guidelines of the Norwegian Ethical and Welfare Board for Animal Research, and the institutional review board approved the study.

### Human kidney biopsies

Kidney biopsies from female SLE patients with nephritis, and from patients with renal cancer or from a patient with Wegener's granulomatosis, were collected, prepared, and stored as described previously [45]. Baseline data for the SLE patients are presented in Figure 3. The glomeruli from the Wegener kidney were devoid of glomerular chromatin-IgG deposits, and the patient did not produce anti-dsDNA antibodies [55].

### Detection of serum anti-dsDNA antibodies by ELISA

Serum anti-DNA autoantibodies were detected by ELISA as previously described [34], [36], [56].

### Renal mRNA levels of nuclease-encoding genes

RNA extraction, cDNA synthesis and real time PCR were performed as previously described [34]. All reagents and assays were from Applied Biosystems. The primers and probes used are presented in Table 1 and in legend to Figure 1 for Dnase1l1/2/3. Expression levels relative to those of 4 w.o. BALB/c mice were calculated for all groups using the ddCT method.

### Protein extraction

Nuclear and nucleus-depleted lysates were prepared from 20mg pieces of snap-frozen, lyophilized kidney tissue using Pierce Nuclear and Cytoplasmic Extraction reagent kit (Pierce Biotechnology, Rockford, IL,). Total protein was measured, and protein content of the samples normalized using BCA assay (Pierce).

### Radial nuclease diffusion assay

To evaluate nuclease activity within native protein samples, a nuclease radial diffusion assay was performed as described [57], with minor modifications. Briefly, 1µl aliquots of renal lysates or serum samples were loaded in 1mm Ø wells on a 1% agarose gel containing 150 µg/ml calf thymus DNA (Sigma-Aldrich GmbH, Steinheim, Germany) and 1 µg/ml ethidium bromide in DNase reaction buffer (40 mM Tris, pH 7.6, 2 mM CaCl_2_ and 2 mM MgCl_2_). The gel was incubated in a humidified chamber at 37°C for 19 hours and photographed under UV illumination.

Heat inactivation of actin was achieved by incubation of samples in a heating block at 56°C for 10 minutes. Dnase1l3 inhibition was performed by addition of 4-(4,6-dichloro-[1,3,5]-triazin-2-ylamino)-2-(6-hydroxy-3-oxo-3H-xanthen-9-yl)-benzoic acid (DR396; Sigma-Aldrich) to a concentration of 5mM. Actin inhibition was achieved by pre-incubating the samples with 5µg/mL bovine g-actin (Sigma-Aldrich) for 10 minutes.

### DNase zymography

DNA degrading activity by Dnase1 was determined after protein separation in a 10% SDS-polyacrylamide gel containing 100µg/ml heat-denatured salmon sperm DNA (Invitrogen Corp., Carlsbad, CA) as described [58]. For actin inhibition experiments, the gels were incubated for 4 hours at RT in 40mM Tris pH 7.4 containing g-actin at a concentration of 5µ g/mL, and 5µg/mL actin was also added to the Dnase reaction buffer, adapting a previously published protocol [27].

### Western blotting

The renal protein extracts (25µg/lane) were separated using 10% SDS-PAGE gels, transferred onto PVDF membranes, and incubated with goat anti-mouse Dnase1 antibodies or goat anti-human Dnase1l1 antibodies (Santa Cruz Biotechnology, Santa Cruz, CA, USA). Binding was visualized using SuperSignal Chemiluminescent Substrate (Pierce) after incubation with HRP-conjugated anti-goat IgG. Equal loading was confirmed by membrane stripping and reprobing with a rabbit anti-human actin IgG (Sigma-Aldrich).

### *In situ* DNA degradation assay

Four µm thick sections of OCT-embedded kidneys were incubated in DNase reaction buffer (2mM CaCl_2_, 2mM MgCl_2_ in 40mM Tris, pH7.6) for 2h at 37°C. The sections were then fixed with 4% paraformaldehyde and assayed using a fluorescent terminal deoxynucleotidyl transferase dUTP nick end labeling (TUNEL) assay kit (Roche Applied Science, Mannheim, Germany).

### Direct immunofluorescence microscopy

Four µm thick cryosections from murine and human kidneys were blocked for 1h with 10% goat serum and 1% bovine serum albumin (BSA) in phosphate-buffered saline (PBS) followed by 30 min incubation with Alexa Fluor 488-conjugated F(ab')_2_ goat anti-mouse or anti-human IgG (Invitrogen) and overnight washing in 0.05% Tween-20 in PBS at +4°C.

### Transmission electron microscopy (TEM) and co-localization immune electron microscopy (IEM)

TEM and co-localization IEM of murine kidney sections were performed exactly as described by Kalaaji et al. [36], [46].

### Indirect immunofluorescence staining

Four µm sections from mouse kidneys embedded in OCT were blocked for 1 hour with 10% goat serum and 1% BSA in PBS followed by washing with PBS and 30 min incubation with goat anti-mouse Dnase1 or anti-human Dnase1l1 antibody (Santa Cruz Biotechnology) or normal goat IgG (negative control). Slides were washed and incubated for 30 min with Alexa Fluor 488-conjugated F(ab')_2_ anti-goat IgG (Invitrogen). Kidney sections from the same mice were incubated with a buffer containing 10% goat serum, 10% fetal calf serum or with 5% BSA in PBS prior to staining with anti-Dnase1 antibody. No difference in Dnase1 staining intensity was seen between these samples. For the human cryopreserved biopsies, rabbit anti-bovine/human Dnase1 antibody (US Biological, Swampscott, Massachusetts) and Alexa Fluor 488-conjugated F(ab')_2_ anti-rabbit IgG (Invitrogen) were used as first and second antibody, respectively.

### Statistics

Statistics were performed with GraphPad Prism 5 (GraphPad Software, San Diego, CA) using the Mann-Whitney U test for comparison of groups. Differences were considered statistically significant at p<0.05.

## Supporting Information

Figure S1 Electron microscopy examination of immune complex deposition in proteinuric (NZBxNZW)F1 mice. Kidney morphology was further studied on ultrathin kidney sections by transmission electron microscopy (A, C, E) to define loci for deposition of electron dense structures, and by co-localization IEM (B, D, F) to detect in vivo-bound IgG (traced by 5nm gold), and chromatin deposits (traced by a monoclonal anti-dsDNA antibody added in vitro to the sections and stained by 10nm gold). In a 28 w.o. B/W mouse with mild proteinuria and sub-normal level of renal Dnase1 activity, the bound anti-dsDNA mAb co-localized with autoantibodies in EDS in the mesangial matrix (Fig. 4A and 4B demonstrate mesangial matrix-associated EDS by TEM, while the anti-dsDNA mAb added to the section in vitro co-localized with in vivo-bound IgG strictly confined to EDS as demonstrated by co-localization IEM, respectively). In a 35 w.o. proteinuric (+3) B/W mouse with low renal Dnase1 activity, the immune complex deposits were observed as EDS in glomerular capillary walls and mesangial matrix by TEM (C). Co-localization IEM demonstrated that these EDS contained IgG molecules and targets for the anti-dsDNA mAb (D). In a 20 w.o. pre-nephritic B/W mouse, TEM (E) revealed normal glomeruli, while co-localization IEM (F) revealed circulating chromatin-containing immune complexes within glomerular capillary lumen (F, enlarged panel), but no immune complexes were associated with membranes or the mesangial matrix. BALB/c mice had normal kidney morphology and no immune complexes were detected by TEM or co-localization IEM (data not shown). In D, it is demonstrated that the anti-dsDNA mAb, added to the sections and traced by 10 nm gold, bound to nuclear DNA.
(3.00 MB TIF)

Figure S2 Phase contrast and indirect immunofluorescence analyses of Dnase1 staining on pre-nephritic and nephritic (NZBxNZW)F1 kidneys. Cryosections of B/W kidneys were analysed by phase-contrast and indirect immunofluorescence using an anti-Dnase1 antibody followed by an Alexa488-conjugated F(ab')2 anti-IgG antibody to stain for Dnase1. The images were taken using identical exposure settings, and were obtained at 200× magnification. Phase-contrast micrographs and corresponding Dnase1 stainings are shown for a pre-nephritic B/W mouse (20 weeks old; panel A), a mouse with mesangial nephritis (panel B) and a mouse with membrano-proliferative nephritis (panel C). Glomeruli have been marked by circles for clarity. As is evident from the figure, Dnase1 is expressed in tubular and glomerular cells, and both compartments loose their Dnase1 expression upon progression of lupus nephritis into end-stage organ disease.
(3.00 MB TIF)

Figure S3 Phase contrast and indirect immunofluorescence analyses of Dnase1 staining of kidney biopsies from patients with lupus nephritis. Cryosections of the kidneys were analysed by phase contrast and indirect immunofluorescence using an anti-Dnase1 antibody followed by an Alexa488-conjugated F(ab')2 anti-IgG antibody to stain for Dnase1. The images were taken using identical exposure settings at 200× magnification. Corresponding phase-contrast micrographs and Dnase1 immunostainings are shown for a patient with mild mesangial (A) and membrano-proliferative (B) lupus nephritis. Glomeruli have been marked by circles for clarity.
(3.00 MB TIF)

Figure S4 Indirect immunofluorescence analyses of histologically normal kidneys and biopsies from patients with Wegener granulomatosus and lupus nephritis. The renal cryosections were immunostained with rabbit anti-Dnase1 antibody followed by an Alexa488-conjugated F(ab')2 anti-IgG antibody. The images were obtained at 400× magnification using identical exposure settings. Strong staining was visible in histologically normal kidneys (A–C). Comparable levels of staining were present in kidneys from a patient with Wegeners granulomatosis (D) and from mesangial lupus nephritis (E), whereas Dnase1 staining was almost undetectable in kidneys from patients with membrano-proliferative lupus nephritis (F–H).
(3.00 MB TIF)
