# Supplementary figures and images for: Renal Dnase1 Enzyme Activity and Protein Expression Is Selectively Shut Down in Murine and Human Membranoproliferative Lupus Nephritis
Source: PLoS One. 2010 Aug 10;5(8):e12096. doi: 10.1371/journal.pone.0012096 (PMC2938370; doi:10.1371/journal.pone.0012096)

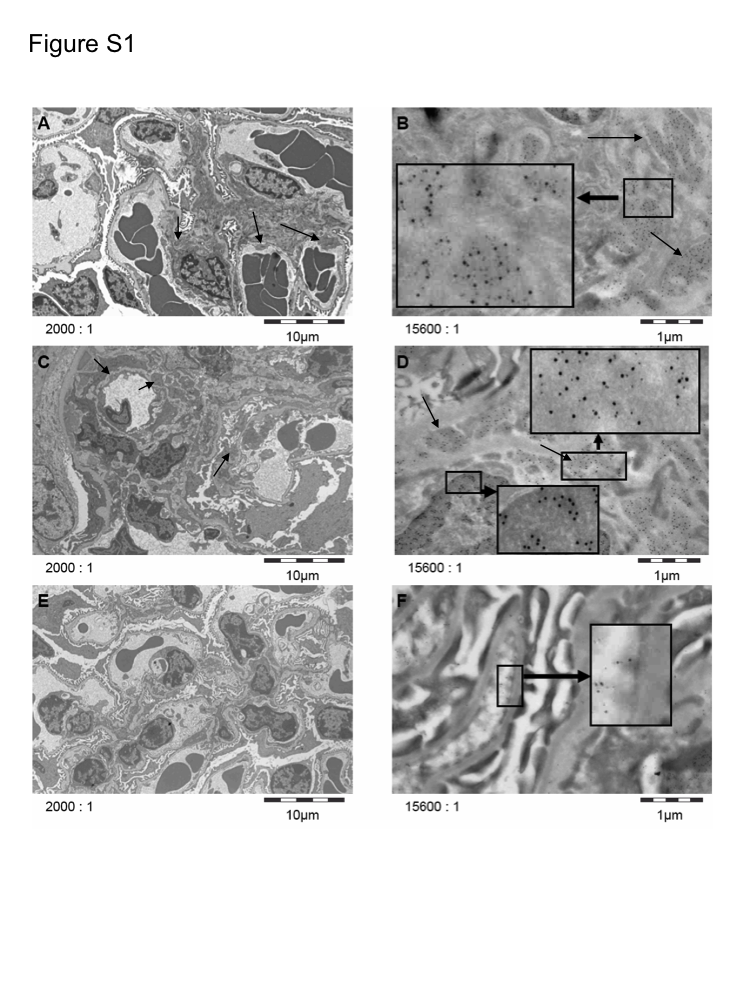

Supplement: Figure S1 — Electron microscopy examination of immune complex deposition in proteinuric (NZBxNZW)F1 mice. Kidney morphology was further studied on ultrathin kidney sections by transmission electron microscopy (A, C, E) to define loci for deposition of electron dense structures, and by co-localization IEM (B, D, F) to detect in vivo-bound IgG (traced by 5nm gold), and chromatin deposits (traced by a monoclonal anti-dsDNA antibody added in vitro to the sections and stained by 10nm gold). In a 28 w.o. B/W mouse with mild proteinuria and sub-normal level of renal Dnase1 activity, the bound anti-dsDNA mAb co-localized with autoantibodies in EDS in the mesangial matrix (Fig. 4A and 4B demonstrate mesangial matrix-associated EDS by TEM, while the anti-dsDNA mAb added to the section in vitro co-localized with in vivo-bound IgG strictly confined to EDS as demonstrated by co-localization IEM, respectively). In a 35 w.o. proteinuric (+3) B/W mouse with low renal Dnase1 activity, the immune complex deposits were observed as EDS in glomerular capillary walls and mesangial matrix by TEM (C). Co-localization IEM demonstrated that these EDS contained IgG molecules and targets for the anti-dsDNA mAb (D). In a 20 w.o. pre-nephritic B/W mouse, TEM (E) revealed normal glomeruli, while co-localization IEM (F) revealed circulating chromatin-containing immune complexes within glomerular capillary lumen (F, enlarged panel), but no immune complexes were associated with membranes or the mesangial matrix. BALB/c mice had normal kidney morphology and no immune complexes were detected by TEM or co-localization IEM (data not shown). In D, it is demonstrated that the anti-dsDNA mAb, added to the sections and traced by 10 nm gold, bound to nuclear DNA. (3.00 MB TIF) [file pone.0012096.s001.tif]

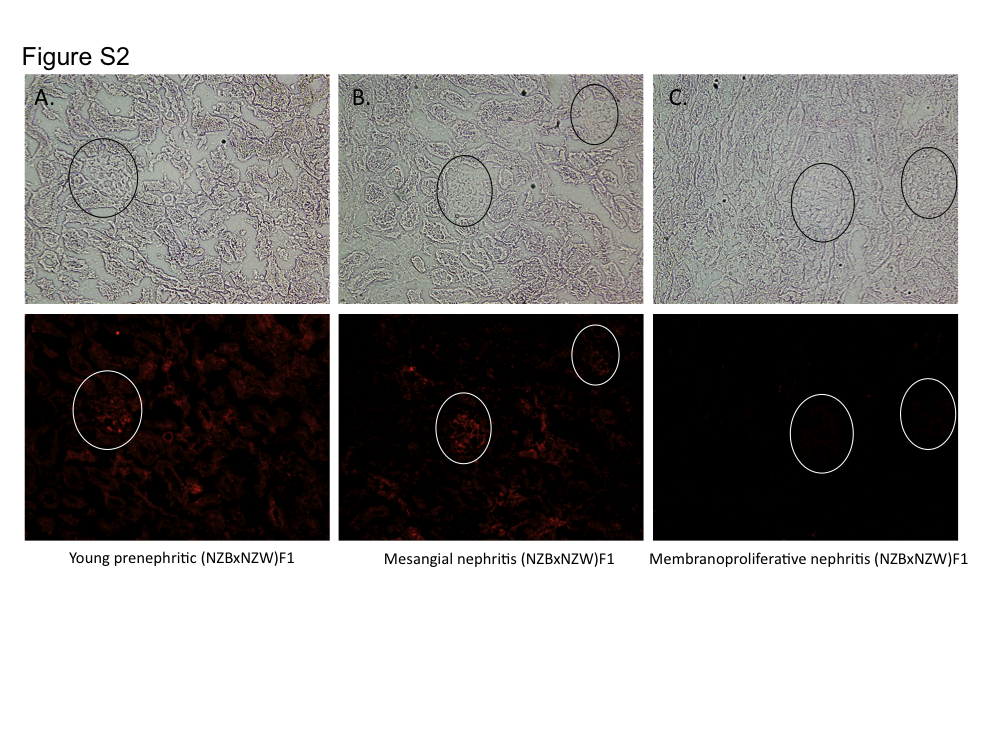

Supplement: Figure S2 — Phase contrast and indirect immunofluorescence analyses of Dnase1 staining on pre-nephritic and nephritic (NZBxNZW)F1 kidneys. Cryosections of B/W kidneys were analysed by phase-contrast and indirect immunofluorescence using an anti-Dnase1 antibody followed by an Alexa488-conjugated F(ab')2 anti-IgG antibody to stain for Dnase1. The images were taken using identical exposure settings, and were obtained at 200× magnification. Phase-contrast micrographs and corresponding Dnase1 stainings are shown for a pre-nephritic B/W mouse (20 weeks old; panel A), a mouse with mesangial nephritis (panel B) and a mouse with membrano-proliferative nephritis (panel C). Glomeruli have been marked by circles for clarity. As is evident from the figure, Dnase1 is expressed in tubular and glomerular cells, and both compartments loose their Dnase1 expression upon progression of lupus nephritis into end-stage organ disease. (3.00 MB TIF) [file pone.0012096.s002.tif]

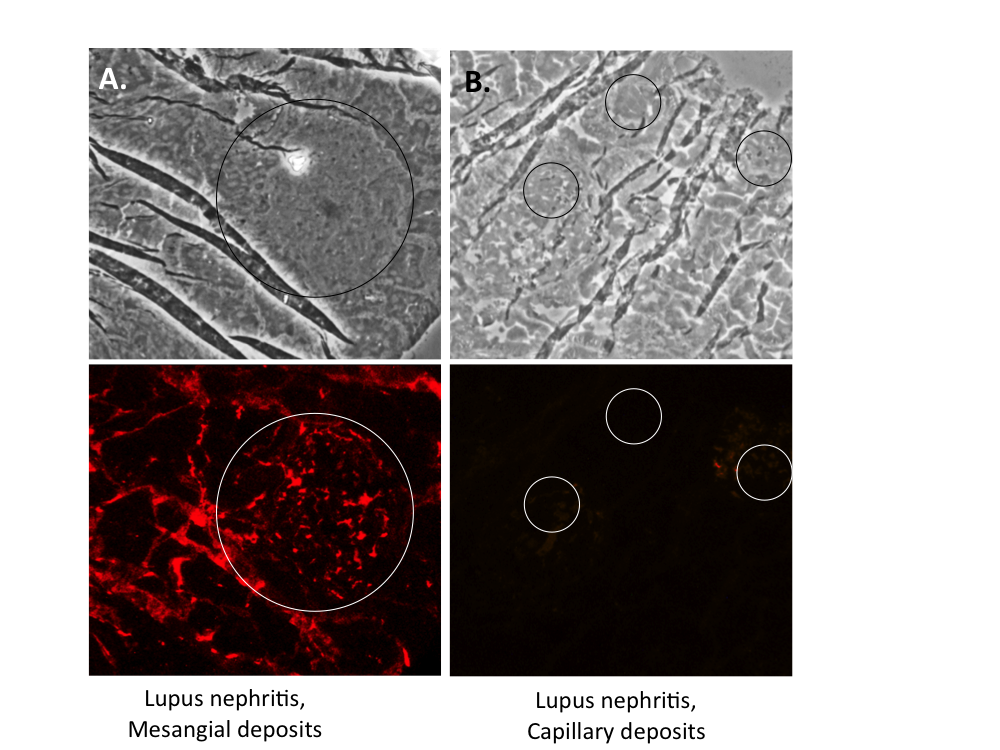

Supplement: Figure S3 — Phase contrast and indirect immunofluorescence analyses of Dnase1 staining of kidney biopsies from patients with lupus nephritis. Cryosections of the kidneys were analysed by phase contrast and indirect immunofluorescence using an anti-Dnase1 antibody followed by an Alexa488-conjugated F(ab')2 anti-IgG antibody to stain for Dnase1. The images were taken using identical exposure settings at 200× magnification. Corresponding phase-contrast micrographs and Dnase1 immunostainings are shown for a patient with mild mesangial (A) and membrano-proliferative (B) lupus nephritis. Glomeruli have been marked by circles for clarity. (3.00 MB TIF) [file pone.0012096.s003.tif]

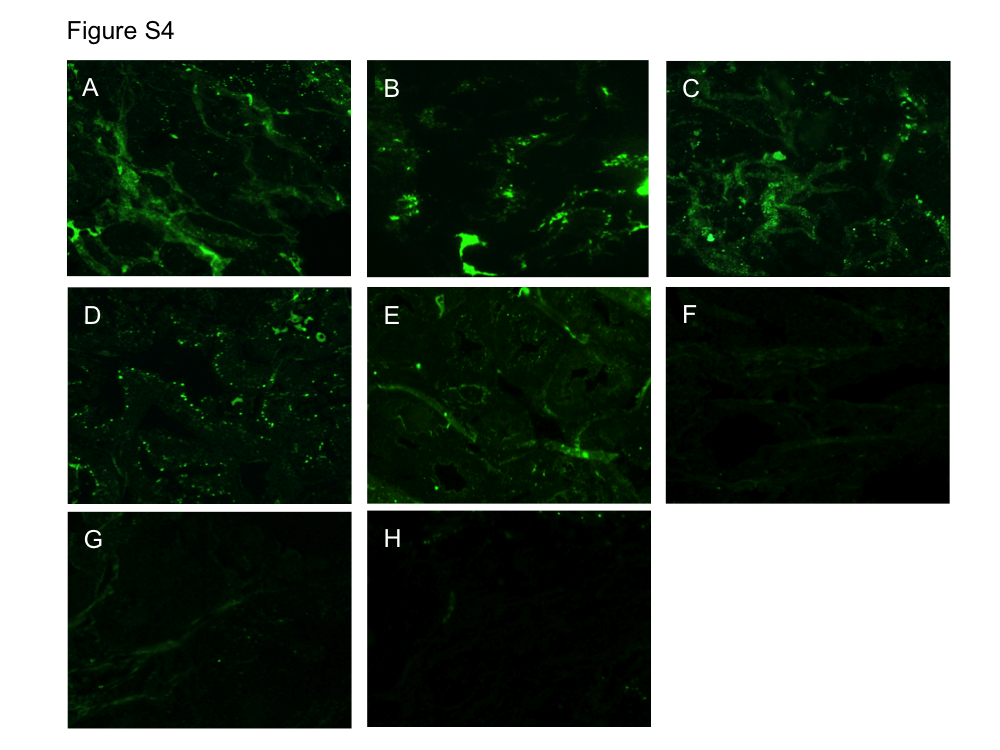

Supplement: Figure S4 — Indirect immunofluorescence analyses of histologically normal kidneys and biopsies from patients with Wegener granulomatosus and lupus nephritis. The renal cryosections were immunostained with rabbit anti-Dnase1 antibody followed by an Alexa488-conjugated F(ab')2 anti-IgG antibody. The images were obtained at 400× magnification using identical exposure settings. Strong staining was visible in histologically normal kidneys (A–C). Comparable levels of staining were present in kidneys from a patient with Wegeners granulomatosis (D) and from mesangial lupus nephritis (E), whereas Dnase1 staining was almost undetectable in kidneys from patients with membrano-proliferative lupus nephritis (F–H). (3.00 MB TIF) [file pone.0012096.s004.tif]
